# Oral Delivery of Psoralidin by Mucoadhesive Surface-Modified Bilosomes Showed Boosted Apoptotic and Necrotic Effects against Breast and Lung Cancer Cells

**DOI:** 10.3390/polym15061464

**Published:** 2023-03-15

**Authors:** Rana Ahmed Youness, Abdulaziz Mohsen Al-Mahallawi, Farah Haytham Mahmoud, Hind Atta, Maria Braoudaki, Sherif Ashraf Fahmy

**Affiliations:** 1 Biology and Biochemistry Department, School of Life and Medical Sciences, University of Hertfordshire Hosted by Global Academic Foundation, Cairo 11835, Egypt; 2 Department of Pharmaceutics and Industrial Pharmacy, Faculty of Pharmacy, Cairo University, Cairo 12613, Egypt; 3 School of Life and Medical Sciences, University of Hertfordshire Hosted by Global Academic Foundation, New Administrative Capital, Cairo 11835, Egypt; 4 Clinical, Pharmaceutical, and Biological Science Department, School of Life and Medical Sciences, University of Hertfordshire, Hatfield AL10 9AB, UK; 5 Chemistry Department, School of Life and Medical Sciences, University of Hertfordshire Hosted by Global Academic Foundation, Cairo 11835, Egypt

**Keywords:** psoralidin, chitosan, bilosomes, oral delivery, apoptosis, breast cancer, lung cancer

## Abstract

This study aims to design and optimize chitosan-coated bilosomal formulations loaded with psoralidin (Ps-CS/BLs) with improved physicochemical properties, oral bioavailability, and boosted apoptotic and necrotic effects. In this regard, uncoated bilosomes loaded with Ps (Ps/BLs) were nanoformulated using the thin-film hydration technique using different molar ratios of phosphatidylcholine (PC), cholesterol (Ch), Span 60 (S60), and sodium deoxycholate (SDC) (1:0.4:0.2:0.125, 1:0.4:0.2:0.25, and 1:0.4:0.2:0.5, respectively). The best-optimized formulation with respect to size, PDI, zeta potential, and EE% was selected and then coated with chitosan at two different concentrations (0.125 and 0.25 *w*/*v*%), forming Ps-CS/BLs. The optimized Ps/BLs and Ps-CS/BLs showed a spherical shape and relatively homogenous size with negligible apparent agglomerations. Additionally, it was demonstrated that coating Ps/BLs with chitosan has significantly increased the particle size from 123.16 ± 6.90 in the case of Ps/BLs to 183.90 ± 15.93 nm in the case of Ps-CS/BLs. In addition, Ps-CS/BLs exhibited higher zeta potential (+30.78 ± 1.44 mV) as compared to Ps/BLs (−18.59 ± 2.13 mV). Furthermore, Ps-CS/BL showed enhanced entrapment efficiency (EE%) of 92.15 ± 7.20% as compared to Ps/BLs (68.90 ± 5.95%). Moreover, Ps-CS/BLs exhibited a more sustained release behavior of Ps compared to Ps/BLs over 48 h, and both formulations were best obeying the Higuchi diffusion model. More importantly, Ps-CS/BLs displayed the highest mucoadhesive efficiency% (74.89 ± 3.5%) as compared to Ps/BLs (26.78 ± 2.9%), indicating the ability of the designed nanoformulation to improve oral bioavailability and extend the residence time inside the gastrointestinal tract upon oral administration. Moreover, upon evaluating the apoptotic and necrotic effects of free Ps and Ps-CS/BLs on human breast cancer cell lines (MCF-7) and human lung adenocarcinoma cell lines (A549), there was a dramatic increase in the percentages of the apoptotic and necrotic cell compared to the control and free Ps. Our findings suggest the possible oral use of Ps-CS/BLs in hampering breast and lung cancers.

## 1. Introduction

**Figure 1:**
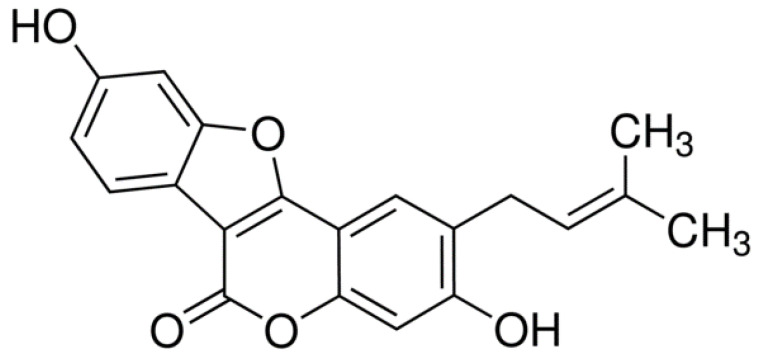
Chemical structure of psoralidin.

Solid tumors, including breast and lung cancers, are the leading reason for mortality all over the globe, with a tremendous annual increase in new incidence cases. Within the coming 20 years, a three-fold increase in new cancer-diagnosed cases is expected [1]. Presently, synthetic chemotherapeutics are widely involved in solid tumor therapy together with radiotherapy and surgical operations. For instance, platinum-based antitumor drugs (such as cisplatin, carboplatin, oxaliplatin, satraplatin, and nedaplatin) [1,2,3,4,5,6], lonidamine, doxorubicin, and methotrexate [7] are commonly used chemotherapeutics in treating solid tumors. Despite their therapeutic anticancer effects, clinicians and patients still suffer from treatment failure of chemotherapeutic agents (even the latest generations) due to their systemic toxicity and cancer cells’ resistance [8]. Thus, phytochemicals developed from natural extracts are recognized as a promising source of more effective and safer anticancer drugs than synthetic ones. Several studies have reported the potential applications of phytochemicals in cancer therapy [9,10,11], such as *Peganum harmala* L. seeds extract [12], [13], betaine (extracted from wheat germ and spinach) [14], fermented Egyptian rice bran (*Oryza sativum*) [15], and ozonated olive oil [16]. In addition, psoralidin (Ps) is a natural phenolic coumarin phytochemical (Figure 1) extracted from the seeds of *Psoralea corylifolia L.* grown in various areas in India, Europe, and Asia [17].

Previous studies reported the broad therapeutic properties of Ps, such as having antimicrobial, antioxidant, antipsoriatic, anti-inflammatory, and antidepressant activities [17]. More importantly, several studies reported the anticancer activities of Ps against numerous cancer cell lines, including breast, esophageal, prostate, colon, and lung cancers [17,18]. Ps exerts its antitumor effects via (i) inducing cellular apoptosis, (ii) modulating autophagy, (iii) promoting DNA aberrations, (iv) decreasing tumor cell proliferation, and (iv) generating oxidative stress [17,18]. Despite the promising anticancer effects of PS, no Ps products are found in the market aiming at cancer treatment. This is due to its hydrophobicity and poor pharmacokinetic properties, limiting its clinical translation [17]. Thus, efforts should be exerted to nanoformulate Ps into a suitable nanosystem which would address the drawbacks hindering its clinical applications. Several nanocarriers are involved in the nanoformulation of different phytochemicals to overcome their limitations and improve their antitumor activities, including vesicular nanocarriers [19,20], polymeric nanoparticles [21,22], supramolecules [23,24], stimuli-responsive delivery systems [25], and mesoporous silica nanoparticles [26]. Vesicular nanocarriers, such as liposomes, niosomes, ethosomes, and bilosomes, are promising lipid-based nanosystems for improving hydrophobic drugs’ therapeutic activities [27]. Bilosomes (BLs) are modified niosomal nanovesicles comprising lipid bilayers containing amphiphilic bile salts (sodium deoxycholate), cholesterol, and surfactant [28]. The inclusion of bile salts was reported to prevent the degradation of the nanosystem in the gastrointestinal tract environment while improving the intestinal membrane’s penetration [28]. The presence of cholesterol is essential to increase the drug’s entrapment efficiency, enhance the rigidity of the bilosomal walls, and promote the intestinal membrane’s permeability [28]. The unique composition of BLs makes them perfect candidates for the oral delivery of various hydrophobic drugs, such as apigenin [29] and quercetin [30].

Chitosan (CS) is a natural biodegradable, biocompatible mucoadhesive cationic polymer widely used to modify the surface of several nanocarriers via the coating process [31]. CS Coating of the surfaces of different nanocarriers is achieved by the electrostatic interaction between the positively charged CS and negatively charged nanocarriers. Coating nanocarriers with CS offers several advantages, including improving stability, extending drug release rates, enhancing membrane penetration, and impeding drug leakage out of the nanovesicles [31].

To the best of our knowledge, no previous studies presented the encapsulation of Ps into nanocarriers to overcome its drawback, except one study that reported the design of poly(methacylic acid-co-methyl methacrylate)/chitosan nanocapsules loaded with Ps aiming at increasing its water solubility and absorption [32].

In this study, we prepared psoralidin (Ps)-loaded bilosomes and chitosan-coated bilosomes loaded with Ps, and the physicochemical characteristics, in vitro release manner, and mucoadhesive efficiency were compared. The selected chitosan-coated Ps bilosomes were assessed for cytotoxic, apoptotic, and necrotic effects in the human breast cancer cell lines (MCF-7) and human lung adenocarcinoma cell line (A549).

## 2. Materials and Methods

### 2.1. Materials

Phosphatidylcholine (PC) and cholesterol were purchased from Corden Pharma (Plankstdt, Germany). Span 60 (S60) and low molecular weight chitosan (CS) were purchased from Biosynth Carbosynth (Berkshire, UK). Dulbecco’s modified Eagle’s medium (DMEM) with 4.5 g/L glucose, 0.05% Trypsin, and phosphate-buffered saline (PBS, pH 7.4). Streptomycin, penicillin, fetal bovine serum, trichloroacetic acid (TCA), Dulbecco’s modified Eagle’s medium (DMEM) SRB, and tris(hydroxymethyl)aminomethane (TRIS) were obtained from Lonza, Basel, Switzerland. Dimethyl sulfoxide (DMSO) was obtained from Serva (Heidelberg, Germany). Annexin V-FITC/PI Apoptosis Detection Kit was purchased from Elabscience (Wuhan, China). MCF-7 and A549 cell lines (Cat No. HTB-22) were purchased from ATCC (Manassas, VA, USA). Sodium deoxycholate, Psoralidin, and all other chemicals were purchased from Sigma-Aldrich (St. Louis, MO, USA).

### 2.2. Preparation of Uncoated and Chitosan-Coated Psoralidin-Loaded Bilosomes

**Scheme 1:**
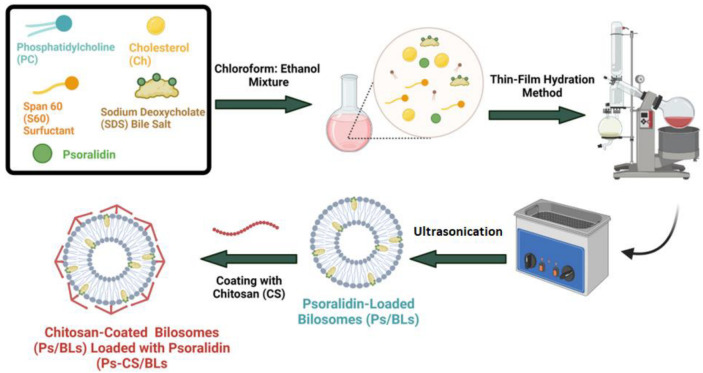
Schematic diagram illustrating the steps involved in the design of surface-modified bilosomes loaded with psoralidin (Ps).

Uncoated psoralidin-loaded bilosomes (Ps/BLs) were developed using the thin-film hydration approach [33] with slight modifications as presented in Scheme 1. Phosphatidylcholine (PC), cholesterol (Ch), Span 60 (S60), Sodium deoxycholate (SDC), and psoralidin (Ps) (in ratios presented in Table 1) were dissolved in a chloroform:ethanol mixture (1:1). A Laboratory 4000 rotary evaporator (Heidolph Instruments, Schwabach, Germany) equipped with a vacuum pump (KNF Neuberger GmbH, Freiburg, Germany) was involved in evaporating the organic solvent under reduced pressure for 1 h at 50 °C, forming a thin film. Then, the dry film was re-hydrated under normal pressure at the same temperature for 1 h using phosphate buffer saline (PBS, pH 7.4). Afterward, The dispersions were ultrasonicated for 10 minutes in a bath sonicator (Elma Hans Schmidbauer, Singen, Germany) for further reductions in particle size. The prepared suspensions were left at room temperature for 45 min before being stored at 4 °C for further investigation. Empty bilosomes were prepared using the same protocol without adding the drug.

Chitosan-coated psoralidin-loaded bilosomes (Ps-CS/BLs) were generated by dropwise addition of two different concentrations of chitosan (0.125 and 0.25%, Table 1) prepared in 1% *v*/*v* glacial acetic acid, using a syringe, to an equal volume of Ps/BLs with continuous stirring (Scheme 1). Then, the mixture was magnetically stirred at 200 rpm for 2 h at room temperature. The 2 prepared formulations were stored at 4 °C for further investigations.

### 2.3. Characterization of the Designed Ps/BLs and Ps-CS/BLs

The particle size, polydispersity index (PDI), and zeta potential of Ps/BLs and Ps-CS/BLs were determined using a Zetasizer Nano ZS equipped with a 10 mW HeNe laser (Malvern Instruments, Worcestershire, UK) at 25 °C. All measurements were carried out in triplicates, and three independent measurements’ standard deviation (SD) was estimated. Surface analysis of Ps/BLs and Ps-CS/BLs was carried out utilizing transmission electron microscopy (TEM) (JEOL-JEM 2100 electron microscope, Musashino, Akishima, Tokyo, Japan) operating at an acceleration voltage of 200 kV. Bilosomes, diluted 1:5 with deionized water, were stained with 2% aqueous phosphotungstic acid and then dried over a carbon-coated copper 200 mesh grid for imaging.

### 2.4. Entrapment Efficiency (EE%)

The indirect method was adopted to study the EE% of the loaded Ps from the different uncoated and coated bilosomal formulations (Table 1) [8]. The free Ps was separated by centrifugation at 12,000× *g* rpm and 4 °C for 3 h (Hermle Z 326 K, Labortechnik GmbH, Wehingen, Germany). Then, the supernatant was separated, and the unloaded Ps was measured employing an ultraviolet-visible UV–Vis spectrophotometer (Carry 60 spectrophotometer, Varian, Palo Alto, CA, USA) at 380 nm. Equation (1) was adopted to estimate the EE%.

(1) $$EE\%=\frac{Initial\;amount\;of\;Ps-the\;amount\;of\;free\;Ps}{Initial\;amount\;of\;Ps}\times 100$$

### 2.5. In Vitro Release Efficiency Percentage (%) of Ps from Ps/BLs and Ps-CS/BLs

The in vitro release percentage (%) was performed to investigate the release behavior of loaded Ps from the different uncoated and coated bilosomal formulations. The dialysis membrane (cutoff molecular weight, 12–14 KD) approach was used for this study. A specific volume of the bilosomes was transferred into a dialysis bag, then dipped into a jar containing phosphate buffer saline (15 mL, PBS, pH 7.4) and Tween 80 (1.5%) to improve the solubility of the released Ps. The jar was placed in a shaking incubator, rotating at 200 rpm at 37 °C. A 1 mL aliquot of the released content was retrieved at designated time intervals and immediately replaced with an isovolumetric fresh buffer solution to achieve sink conditions. The released Ps was quantified using an ultraviolet-visible (UV–Vis) spectrophotometer (Carry 60 spectrophotometer, Varian, Palo Alto, CA, USA) at 380 nm. The release efficiency % was determined using Equation (2).

(2) $$Release\;Efficiency\;\%=\frac{Amount\;of\;released\;Ps}{Initial\;amount\;of\;loaded\;Ps}\times 100$$

The study was conducted in triplicate, and the standard deviation (SD) of three independent measurements has been estimated. Then, it was graphically plotted as the release efficiency (%) versus time.

### 2.6. Release Kinetics Study

The release efficiency (%) values obtained from the in-vitro drug release study were further analyzed using various kinetic models to investigate the Ps release pattern from the uncoated and coated bilosomal formulations. In this regard, five drug-release kinetic models were investigated: zero-order, first-order, Higuchi model, Korsmeyer–Peppas, and Hixson-Crowell kinetic models, as previously described based on the following equations [34,35]:

(3) $$C=K_{o}t$$

(4) Log(100−C)=−Kft2.303

(5) $$C=K_{H}\sqrt{t}$$

(6) $$C=K_{k}t^{n}$$

(7) $$\sqrt[{3}]{W_{0}}-\sqrt[{3}]{W_{t}}=K_{ß}t$$

where *C* is the cumulative % drug released at time *t*, *K*_0_ is the zero-order rate constant, *K_f_* is the first-order rate constant, *K_H_* is the Higuchi constant, *K_K_* is the Korsmeyer–Peppas constant, *n* is the exponent that describes a particular diffusion mechanism, *W*_0_ is the initial amount of drug in the system, *W_t_* is the remaining amount of drug in the system at time *t*, and *K_ß_* is the Hixson-Crowell release constant.

### 2.7. Mucoadhesive Efficiency (ME%)

The mucoadhesive study was conducted by adopting the adsorption techniques using mucin, as described previously, with slight modifications [36]. The unloaded Ps, Ps/BLs, and Ps-CS/BLs were mixed and vortexed with standard mucin solution (0.5 mg/mL) in a molar ratio of 1:1 and then magnetic stirred at room temperature for 2 h. Then, mixtures were centrifuged at 6000× *g* rpm for 2 h, and the supernatant was separated.

Then, mucin concentration was quantified spectrophotometrically, and the concentration of mucin adsorbed was determined according to Equation (3).

(8) $$ME\%=\frac{Initial\;concentration\;of\;mucin-the\;concentration\;of\;free\;mucin}{Initial\;concentration\;of\;mucin}\times 100$$

### 2.8. In Vitro Cell Viability Assay

#### 2.8.1. Cell Culture

Human breast cancer cell lines (MCF-7) and human lung adenocarcinoma cell lines (A549) obtained from American Type Culture Collection (University Boulevard, Manassas, VA 20110) were used in this study. Cancer cell lines were cultured in Dulbecco’s modified Eagle’s medium (DMEM; Lonza, Switzerland) supplemented with 4.5 g/L glucose, 4 mmol/L l-glutamine, 10% fetal bovine serum (FBS), 100 mg/mL of streptomycin, 100 units/mL of penicillin, and MycoZap (1:500; Lonza, Switzerland) at 37 °C in 5% CO_2_ atmosphere. The cells were passaged upon reaching 85-90% confluency, as previously described [37,38].

#### 2.8.2. Sulforhodamine B Colorimetric Assay

The Sulforhodamine B (SRB) cytotoxicity assay was used in order to assess the impact of empty bilosomes, Ps, and Ps-CS/BLs on the cellular viability of MCF-7 and A549 cell lines. In a 96-well plate, 7000 MCF-7 or A549 cells were seeded and incubated in full DMEM for 24 h. Cells were then treated with 100 μL free DMEM containing various concentrations of empty bilosomes, Ps, and Ps-CS/BLs (0.01, 0.03, 0.1, 0.3, 1, 3, 10, 30, 100, and 300 μg/mL). Forty-eight hours post-treatment, cells were fixed using 10% TCA and incubated at 4 °C for 1 h. The TCA solution was then removed, and the cells were washed with distilled water five times. Then, 70 μL SRB solution (0.4% *w*/*v*) was added and incubated in the dark at room temperature for 10 min. Treated wells with SRB were then washed thrice with 1% acetic acid and allowed to air-dry for 24 h. Then, 150 μL of TRIS (10 mM) was added to solubilize the protein-bound SRB stain. Control groups were cancer cells cultured in serum-free media treated with the vehicle for all trials. The absorbance was measured at 540 nm using a BMG LABTECH^®^-FLUOstar Omega microplate reader (Ortenberg, Germany).

### 2.9. Flow Cytometry (Annexin V Apoptosis Assay)

Cellular apoptosis is investigated using an Annexin V-FITC apoptosis detection kit (Abcam, UK) coupled with two fluorescent channels flow cytometry. GraphPad Prism was used to calculate IC_50_ values for the Ps and its bilosomal formulation based on the findings of the cell viability assay. As a result, the IC_50_ values were employed to treat MCF-7 and A549 cells for 48 h. Post-treatment of tested formulations, 100,000 cells were collected by trypsinization and washed twice with ice-cold PBS (pH 7.4). Then, cells were incubated with 0.5 mL of Annexin V-FITC/PI solution for 30 min in the dark at room temperature according to manufacturer protocol. After the staining procedure, cells are injected via ACEA Novocyte™ flow cytometer (ACEA Biosciences Inc., San Diego, CA, USA) and analyzed for FITC and PI fluorescent signals using an FL1 and FL2 signal detector, respectively (λex/em 488/530 nm for FITC and λex/em 535/617 nm for PI). For each sample, 12,000 events are acquired, and positive FITC and/or PI cells are quantified by quadrant analysis. The experiment was repeated thrice, and the obtained data were analyzed using ACEA NovoExpress™ software (ACEA Biosciences Inc., USA).

### 2.10. Statistical Analysis

All presented data are shown as mean ± standard deviation. The unpaired Student’s *t* test (parametric and two-tailed) was used to compare the two groups. One-way ANOVA was used to compare more than two groups. All experiments were performed in triplicates and repeated at least three times. *p*-value < 0.05 was considered statistically significant. All the data were statistically analyzed using GraphPad Prism 5.00 software.

## 3. Results and Discussion

### 3.1. Average Diameters, PDI, Zeta-Potential, and Entrapment Efficiency (EE%)

Different ratios of phosphatidylcholine (PC), cholesterol (Ch), Span 60 (S60), and sodium deoxycholate (SDC) (1:0.4:0.2:0.125, 1:0.4:0.2:0.25, and 1:0.4:0.2:0.5, respectively) were used to design the bilosomes loaded with Ps (Ps/BLs). The best-optimized formulation concerning size, PDI, zeta potential, and EE% was selected for further coating with chitosan.

In this context, the average particle size, PDI, and zeta potential of the different bilosomal formulations were detected by the dynamic light scattering, as presented in Table 1. Ps/BLs fabricated using PC/Ch/S60/SDC in a ratio of 1:0.4:0.2:0.25 (denoted a code of B2 in Table 1) had the lowest average particle size (123.16 ± 6.90 nm), PDI (0.21 ± 0.05), and outstanding zeta potential (−18.59 ± 2.13 mV), as compared to the rest of formulations. The small particle size of Ps/BLs (B2) would enable the passive uptake of the bilosomes in cancer cells with leaky vasculature and poor lymphatic drainage [39]. Furthermore, it was revealed that B2 had the highest EE% (68.90 ± 5.95%) as compared to the other 2 formulations (B1 and B3), as presented in Table 1. This improved EE% of B2 is due to its higher surface-to-volume ratio resulting from its smaller particle size. The high EE% is an essential factor that enhances the anticancer activity of the formulation.

Based on the above findings, the selected optimized formulation (B2) was coated with chitosan at 2 different concentrations (0.125 and 0.25 *w*/*v*%, B2-CS1 and B2-CS2, respectively). A significant increase in particle size, zeta potential, and EE% (*p* < 0.5) was observed (Table 1).

It was revealed that coating B2 formulation with either 0.125 or 0.25 *w*/*v* chitosan (B2-CS1 and B2-CS2, respectively) has significantly increased the particle size (*p* < 0.5) from 123.16 ± 6.90 to 150.18 ±12.42 (B2-CS1) and 183.90 ± 15.93 (B2-CS2) nm, respectively. As previously reported, as the particle size increases above 500 nm, the particles would enter the lymphatic drainage system, while particles below 500 nm transport their payloads inside the cancerous cells via endocytosis. In addition, the high surface area of the nanoparticles (<500 nm) increases the absorption of the loaded drugs [40]. Furthermore, it was shown that the PDI of B2 (0.21) did not increase significantly (*p* < 0.5) upon coating with either concentration of chitosan (0.23 and 0.24, respectively), indicating the homogeneity and narrow size distribution of the designed bilosomal formulations [39]. In addition, coating the B2 with 2 different concentrations of CS has been shown to increase the zeta potential from −18.59 ± 2.13 mV in the case of B2 to +21.89 ± 4.98 and +30.78 ± 1.44 mV in the case of B2-CS1 and B2-CS2, respectively. This indicates the successful coating of the negatively charged Ps/BLs with the cationic chitosan via electrostatic interactions [36]. This high surface positive charge is reported to increase the stability of the bilosomal formulations by minimizing their clumping and particle aggregation [41]. Moreover, the high positive charge of B2-CS2 will enhance the cellular interaction and penetration of the Ps-CS/BLs into cancer cells [42]. Furthermore, our findings showed that coating Ps/BLs with either concentration of chitosan (0.125 or 0.25 *w*/*v*) has significantly increased the EE% from 68.90 ± 5.95% (in the case of B2) to 88.96 ± 8.65 and 92.15 ± 7.20 (in the case of B2-CS1 and B2-CS2, respectively). This increase in EE% is attributed to the use of CS as a coating polymer, which is reportedly to hamper the leakage of entrapped drugs from bilosomes [43].

Since B2-CS2 (Ps-CS/BLs) was the best-optimized formulation regarding particle size, zeta potential, and EE%, it was selected for further experiments compared to B2 (Ps/BLs).

### 3.2. Morphological Features of Ps/BLs and Ps-CS/BLs

**Figure 2:**
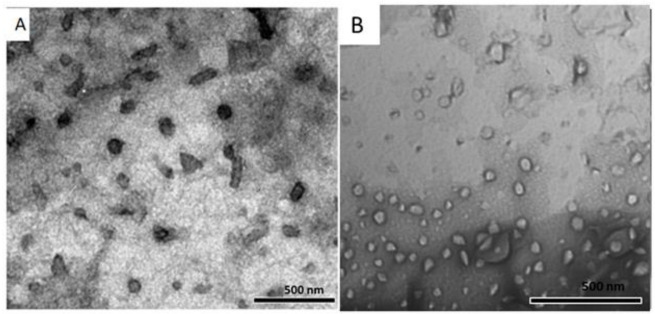
TEM images for (**A**) Ps/BLs and (**B**) Ps-CS/BLs.

TEM was used to study the morphology and surface properties of the optimized uncoated and chitosan-coated bilosomes. As presented in Figure 2, both Ps/BLs and Ps-CS/BLs showed a spherical shape and relatively homogenous size with negligible apparent agglomerations. In addition, our findings revealed that the spherical shape of bilosomes has not been affected upon coating with CS. Furthermore, Figure 2B shows the thin CS layer coating Ps/BLs.

### 3.3. In Vitro Release Efficiency (%) Study

**Figure 3:**
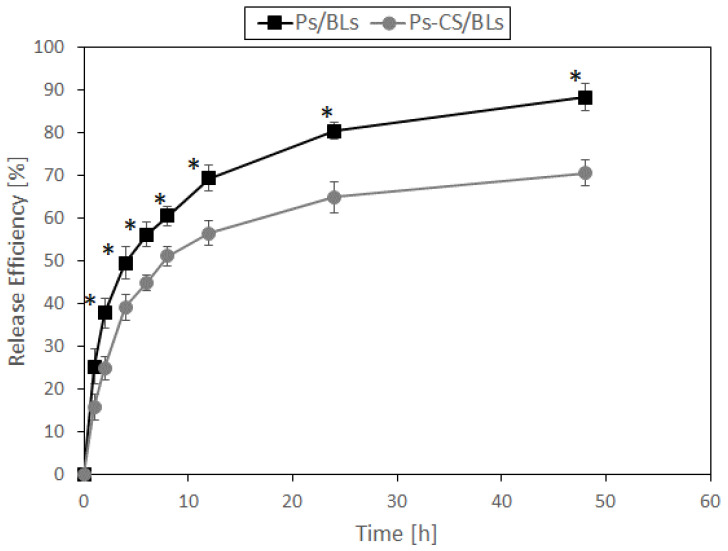
Release efficiency% of Ps from Ps/BLs and Ps-CS/BLs at 37 °C, into phosphate buffer (pH 7.4). Data represent means ± SD (n = 3). *p*-values < 0.05 were considered statistically significant, as indicated by * for comparing Ps-CS/BLs to Ps/BLs.

Figure 3 depicts the release % of unloaded Ps from either Ps/BLs or Ps-CS/BLs into the PBS medium at 37 °C. For both formulations, Ps exhibited a biphasic release manner over 48 h. In the first 2 h, rapid diffusion of Ps from the dialysis bag took place from Ps/BLs (37.78 ± 3.45%) and Ps-CS/BLs (24.89 ± 2.76%). This is followed by a sustained release behavior, where 69.34 ± 2.96 and 56.46 ± 2.8% were effluxed from the dialysis bag after 12 h and 88.29 ± 3.12 and 70.59 ± 30 were effluxed from the dialysis bag after 48 h in the case of Ps/BLs and Ps-CS/BLs, respectively. The initial rapid release of Ps from both bilosomal formulations is attributed to the presence of Ps on the bilosomal outer surface. In addition, the nano-scale particle sizes of both formulations (<185 nm) increased the particles’ effective surface area, expanding the interaction points of Ps with the dissolution medium [44]. The later sustained release manner of Ps from both formulations is due to the entrapment of the payload in the inner center of the bilosomes, which is then slowly released by erosion and diffusion [45]. Moreover, the release study findings revealed significantly slower release rates of Ps from Ps-CS/BLs as compared to the uncoated Ps/BLs (*p* < 0.5). This behavior is due to the presence of CS coating polymer over the bilosomal outer surface, which retards the diffusion of Ps, which has to pass two layers (the bilosomal bilayers and the CS layer) in order to reach the dissolution medium. These findings show the ability of CS coating to extend the release of drugs from the bilosomes, which is idyllic for designing sustained oral delivery systems for cancer treatment [36].

### 3.4. Release Kinetics Study

**Figure 4:**
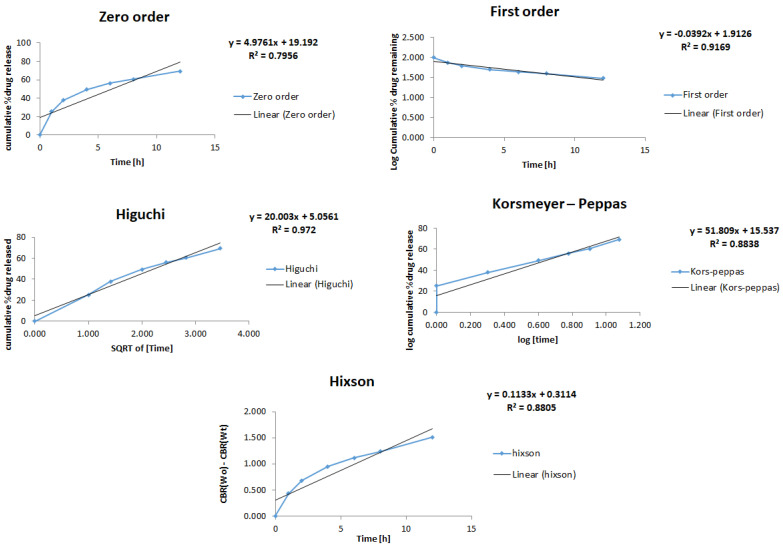
Release kinetics of Ps from Ps/BLs fitted to five kinetic models.

**Figure 5:**
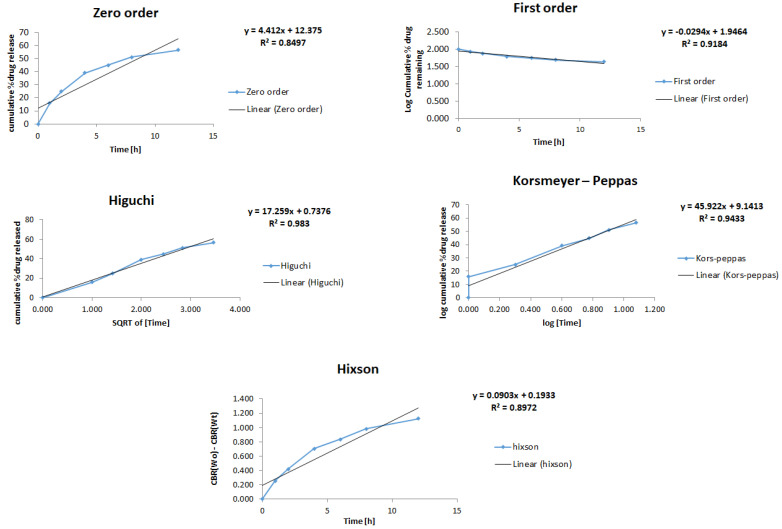
Release kinetics of Ps from Ps-CS/BLs fitted to five kinetic models.

The release data of Ps from Ps/BLs and Ps-CS/BLs (Figure 4 and Figure 5) were fitted to five different models (zero-order, First order, Higuchi, Korsmeyer–Peppas, and Hixson models) in order to determine the drug release profile kinetics from either formulation. Table 2 presents the kinetic release parameters and regression coefficients computed from the five kinetic models subjected to this investigation. As presented in Figure 4 and Figure 5 and Table 2, the correlation coefficient (*R*^2^) values calculated by fitting in each of the five kinetic models indicate that the release of Ps from either Ps/BLs or Ps-CS-BLs is best obeying the Higuchi diffusion model. It has been shown that the highest correlation coefficients were obtained with the Higuchi model (*R^2^* values of 0.97 and 0.98 in the case of Ps-BLs and Ps-CS/BLs, respectively. The Higuchi diffusion kinetic model described drug release as a controlled diffusion phenomenon relying on Fick’s law (square root time-dependent) from different matrices [35]. This could be explained by the fact that the drug dissolves firstly within the lipid bilayer followed by diffusion outside, which is the rate-limiting step. These findings are consistent with several previous studies that demonstrated the fitting of drug release from vesicular systems to the Higuchi model. Glavas-Dodov, Marija, et al. demonstrated that the release of 5-fluorouracil from topical liposome gels for anticancer treatment fitted to the Higuchi model [46]. Another study revealed that the release of silver sulfadiazine from cubosomal formulations followed the Higuchi diffusion model [47].

### 3.5. Mucoadhesive Efficiency (ME%)

**Figure 6:**
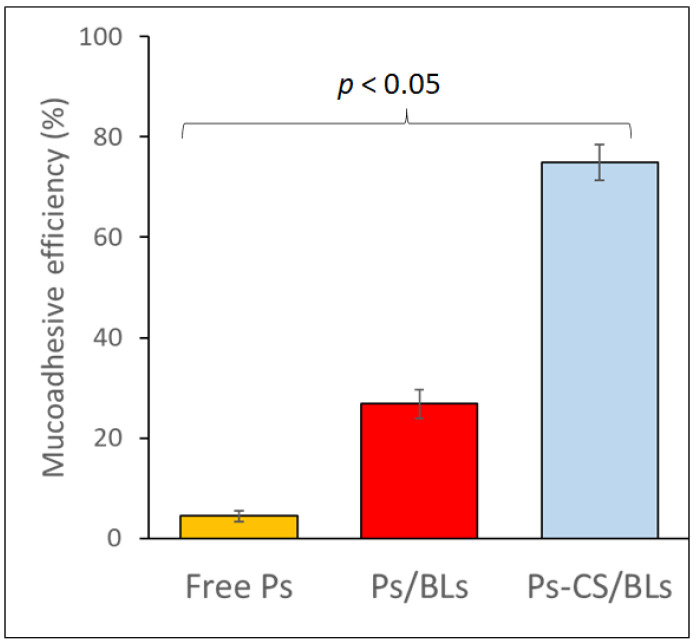
Mucoadhesive efficiency percentage (ME%) of free Ps, Ps/BLs, and Ps-CS/BLs.

The adsorption approach evaluated the mucoadhesive efficiencies of free Ps and the optimized bilosomal formulations (Ps/BLs and Ps-CS/BLs), and the results are presented in Figure 6. A high mucin concentration on the sample’s surface under investigation demonstrates a higher binding capacity [36]. As illustrated in Figure 6, Ps-CS/BLs exhibited the highest ME% (74.89 ± 3.5%) as compared to free Ps (4.48 ± 1.09%) and Ps/BLs (26.78 ± 2.9%) (*p* < 0.5). The higher ME% of Ps-CS/BLs compared to the other two samples is due to the cationic CS layer coating the bilosomal formulation, which binds the negatively charged mucin via electrostatic interaction. These findings align well with the zeta potential, TEM, EE%, and in vitro release study findings.

Our findings clearly indicate the ability of the chitosan-coated bilosomal formulation to bind the anionic mucin found in the intestine. This notable mucoadhesive capability enhances oral bioavailability, extends the residence time inside the gastrointestinal tract, and improves the therapeutic efficiency of Ps upon oral administration [48].

### 3.6. In Vitro Cell Viability Assay

**Figure 7:**
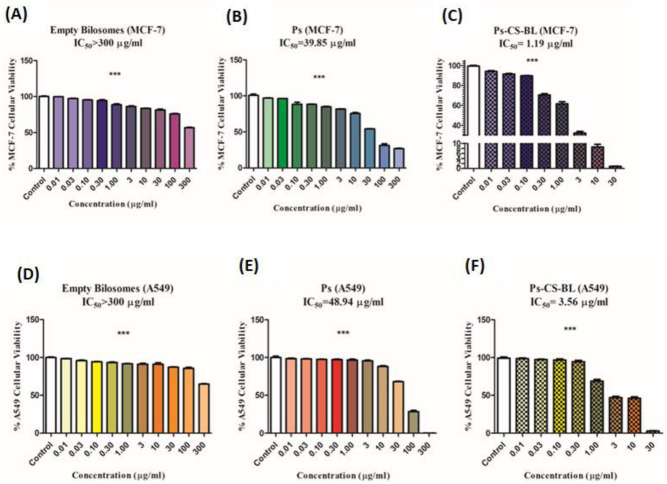
Evaluating the cytotoxicity of (**A**) empty bilosomes, (**B**) Ps/BLs, and (**C**) Ps-CS/BLs in MCF-7 cells, and (**D**) empty bilosomes, (**E**) Ps/BLs, and (**F**) Ps-CS/BLs in A549 cells using SRB assay, at several concentrations ranging from 0.01 to 300 µg/mL. An overall statistically significant decrease in cell viability was detected with Ps-CS/BLs as compared to free Ps in both cancer cells (*p* < 0.05). Untreated cells were utilized as negative control (considered as 100%). (*** *p* < 0.05 compared with the control group).

Since Ps-CS/BLs demonstrated a small particle size, the highest positive zeta potential, EE%, sustained release behavior, and ME%, they were chosen for the cell viability evaluation. In this regard, the effect of empty bilosomes, Ps, and Ps-CS/BLs was assessed on breast adenocarcinoma cells (MCF-7) and lung cancer cells (A-549) involving SRB assay. It was found that the empty bilosomes (host control) showed relatively non-cytotoxic effects in both cancer cell lines with IC_50_ values above 300 µg/ml, as shown in Table 3 and Figure 7A,D. On the other hand, the Ps-CS/BLs formulation exhibited a significantly higher (*p* < 0.5) in vitro cytotoxic activity against MCF-7 (Figure 7B,C) and A-549 (Figure 7E,F) cancer cells, with IC_50_ values of 1.19 ± 0.24, and 3.56 ± 0.36 µg/mL, respectively; this is as compared to free Ps (IC_50_ of 39.85 ± 1.1 and 48.94 ± 1.3 µg/mL, respectively), as presented in Table 3.

Ps was reported to exert its anticancer effect by inducing cellular apoptotic pathways, modifying autophagy, encouraging DNA aberrations, or reducing tumor cell proliferation [17,18]. Several previous studies reported the anticancer effects of Ps against several solid malignancies, including prostate [49], colon [50], liver [51], and cervical [52] cancers. However, our study is considered one of the fewest studies unraveling the anticancer activity of Ps on human breast and lung cancer cell lines. Only a recent study supported the anticancer activity of Ps in the murine BC mice model [53]. Yet, our study might be considered the first to unravel the potency of Ps in repressing oncogenic hallmarks of MCF-7 and A549 cells. In addition, our study reported the enhancement of the anticancer activities of Ps against both cancer cell lines subjected to the current investigation upon its loading into chitosan-coated bilosomes (Ps-CS-BLs). This is attributed to increasing the hydrophilicity of Ps upon its encapsulation into bilosomes; thus, a much higher concentration of the drug reaches the intended site of action [54]. Moreover, the nano-scale particle size and the high ME% of Ps-CS/BLs resulting from CS coating improve the bioavailability of the drug and its accumulation inside cancer cells by loosening the constricted junctions of the cells. Finally, the cationic CS surrounding the bilosomal formulation could interact with the negatively charged tumor cells [55,56]. Notably, previous studies have reported the differential cytotoxic effects of Ps on cancer cells without harming non-cancerous ones. For instance, Pal et al. reported Ps’ anti-proliferative and apoptotic effects against cadmium-transformed prostate epithelial cells (CTPE) without conferring any cytotoxicity against the normal prostate epithelial cells [57].

### 3.7. Flow Cytometry and Cell Apoptosis Assay

Though it was apparent that entrapping Ps in chitosan-coated bilosomes has enhanced its cytotoxic effects on MCF-7 and A549 cancer cells, it was essential to explore the modality of cell death in response to free Ps and Ps-CS/BLs. In this regard, MCF-7 and A549 cells were treated for 48 h with the IC_50_ concentrations computed by the SRB assay. Afterward, the percentages of apoptotic and necrotic cells were determined utilizing Annexin/PI staining, measured by flow cytometry analysis, as previously detailed.

**Figure 8:**
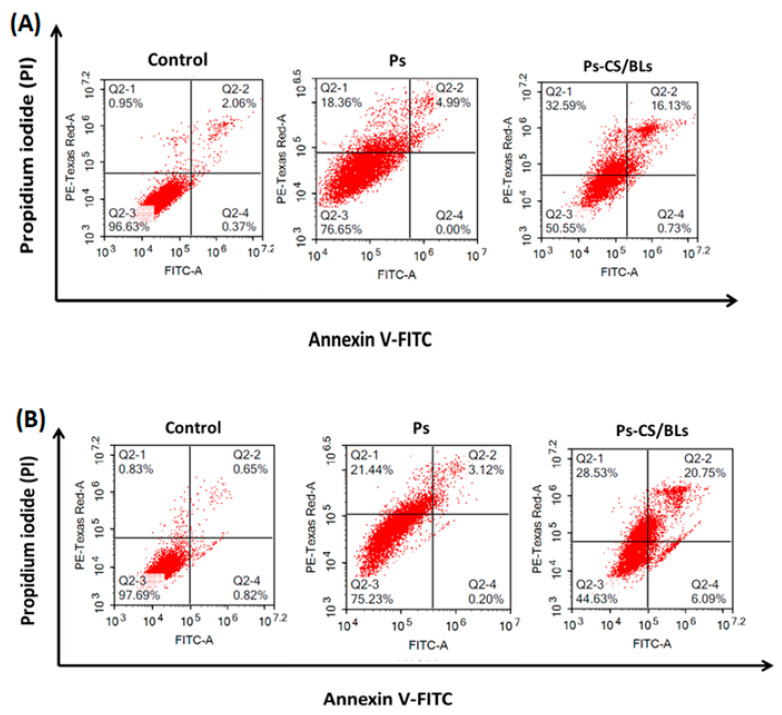
Apoptotic and necrotic effects of free and loaded Ps on (**A**) MCF-7 and (**B**) A549 cells. This figure represents a representative figure from flow cytometric analysis experiments and the apoptotic assay. This experiment was performed in triplicates.

Our findings showed that free Ps could increase the percentage of apoptotic cells by 5% and the percentage of necrotic cells by 18.36% compared to the control in the case of MCF-7. At the same time, it increases the percentage of apoptotic cells by 3.12% and the percentage of necrotic cells by 21.44% compared to the control in the case of A549 (Figure 8A,B). Notably, when cancer cells were treated with the IC_50_ concentration of Ps-CS/BLs, the percentage of apoptotic cells augmented to 16.13% in the case of MCF-7 and 20.75% in the case of A549 compared to the control. Similarly, the percentage of necrotic cells increased to 32.59% in the case of MCF-7 and 28.53% in the case of A549 compared to the control (Figure 8A,B). Our findings align well with the previous studies reporting that Ps exerts its anticancer activity by inducing the apoptotic pathways [17,18]. Captivatingly, and to the best of our knowledge, there have been no previous studies evaluating the apoptotic and necrotic effects of Ps in human breast and lung cancers and comparing it to a nanocarrier loading Ps in which Ps was entrapped in chitosan-coated bilosomal nanoformulation (Ps-CS/BLs). In addition, our findings align with previous studies that reported the chitosan effects in inducing apoptosis mainly by activating caspase-3 [58] or FAS/FAS-L [59].

## 4. Conclusions

The in vitro oral bioavailability and anticancer effects of the hydrophobic psoralidin were improved via loading into chitosan-coated bilosomal nanoformulation (Ps-CS/BLs). In this context, the surface-modified bilosomes loaded with Ps were developed by the thin-film hydration approach followed by coating with cationic chitosan. The designed Ps/BLs and Ps-CS/BLs were assessed in terms of size, zeta potential, entrapment efficiency (EE%), mucoadhesive efficiency (ME%), and release kinetics. Both nanoformulations exhibited nano-sized and monodispersed particles, outstanding zeta potential, and high entrapment efficiencies. Both formulations were shown to obey the Higuchi diffusion kinetic model, but the Ps-CS/BLs exhibited a more sustained release behavior over 48 h and a higher ME%. Additionally, the Ps-CS/BLs showed the highest cytotoxic activities against human breast cancer cell lines (MCF-7) and human lung adenocarcinoma cell line (A549) as compared to free Ps. Moreover, the Ps-CS/BLs exhibited the highest percentages of apoptosis and necrosis when tested on MCF-7 and A549 cells. Collectively, these findings reveal that chitosan-coated bilosomes encapsulating Ps could be a promising tactic for conquering breast and lung cancers via the oral route.

## Figures and Tables

**Table 1:** The particle size, PDI, zeta-potential, and EE% of different uncoated and chitosan-coated bilosomal formulas. All measurements were carried out in triplicate, and the data are expressed as means ± standard deviations.

Code	Molar Ratio			Concentration (mM)	Concentration (% *w/v*)	Size (nm)	PDI	Zeta Potential (mV)	EE%
	PC	Ch	S60	SDC	CS				
**B1**	**1**	**0.4**	**0.2**	0.125	-	118.29 ±10.23	0.12 ± 0.04	−12.34 ± 1.93	50.10 ± 3.94
**B2**	1	0.4	0.2	0.25	-	123.16 ± 6.90	0.21 ±0.05	−18.59 ± 2.13	68.90 ± 5.95
**B3**	1	0.4	0.2	0.5	-	175.34 ± 9.29	0.29 ± 0.09	−19.34±3.61	59.17 ± 4.23
**B2-CS1**	1	0.4	0.2	0.25	0.125	150.18 ±12.42	0.23 ± 0.2	+21.89 ± 4.98	88.96 ± 8.65
**B2-CS2**	1	0.4	0.2	0.25	0.25	183.90 ± 15.93	0.24 ±0.3	+30.78 ± 1.44	92.15 ± 7.20

Abbreviations: PC: phosphatidylcholine, Ch: cholesterol, S60: Span 60, SDC: sodium deoxycholate, and CS: Chitosan.

**Table 2:** Rate constants and correlation coefficients (*R*^2^) of Ps released from Ps/BLs and Ps-CS/BLs computed by fitting into five different models (zero-order, First order, Higuchi, Korsmeyer–Peppas, and Hixson models).

Models	Ps/BLs	Ps-CS/BLs
**Zero-order**		
*k*_0_	4.98	4.41
*R* ^2^	0.79	0.85
**First order**		
*k_f_*	0.04	0.03
*R* ^2^	0.92	0.92
**Higuchi**		
*k_H_*	20.00	17.26
*R* ^2^	0.97	0.98
**Korsmeyer–Peppas**		
n	51.81	45.92
*R* ^2^	0.88	0.94
**Hixson**		
κ t	0.11	0.09
*R* ^2^	0.88	0.89

**Table 3:** IC_50_ Values for empty bilosomes, Free Ps, and Ps-CS/BLs.

Cells	IC_50_ Values (µg/mL)		
	Empty BLs	Ps	Ps-CS/BLs
**MCF-7**	>300	39.85 ± 1.1	1.19 ± 0.24
**A-549**	>300	48.94 ± 1.3	3.56 ± 0.36

## Data Availability

The data presented in this study are available upon request from the corresponding author.
